# CO Preferential Photo-Oxidation in Excess of Hydrogen in Dark and Simulated Solar Light Irradiation over AuCu-Based Catalysts on SBA-15 Mesoporous Silica-Titania

**DOI:** 10.3390/ma11071203

**Published:** 2018-07-13

**Authors:** Isabel Barroso-Martín, Antonia Infantes-Molina, Aldo Talon, Loretta Storaro, Elena Rodríguez-Aguado, Enrique Rodríguez-Castellón, Elisa Moretti

**Affiliations:** 1 Departamento de Química Inorgánica, Cristalografía y Mineralogía (Unidad Asociada al ICP-CSIC), Facultad de Ciencias, Universidad de Málaga, Campus de Teatinos, 29071 Málaga, Spain; isabel.barroso@uma.es (I.B.-M.); aguadoelena5@gmail.com (E.R.-A.); castellon@uma.es (E.R.-C.); 2 Dipartimento di Scienze Molecolari e Nanosistemi, Università Ca’ Foscari Venezia, National Interuniversity Consortium of Materials Science and Technology (INSTM) Venice Research Unit, Via Torino 155/B, Mestre, 30172 Venezia, Italy; aldair@unive.it (A.T.); storaro@unive.it (L.S.)

**Keywords:** CO preferential oxidation, CO-PROX, photocatalysis, solar light response, SBA-15 mesoporous silica, Ti-SBA-15, gold nanoparticles

## Abstract

In this work, SBA-15 silica and silica-titania have been used as supports for photocatalysts based on AuCu alloy (Au:Cu = 1) to be used in the preferential oxidation of CO (CO-PROX) in excess of hydrogen at room temperature and atmospheric pressure both in the dark and under simulated solar light irradiation. To study their textural, structural, chemical and optical properties, the samples were characterized by X-ray diffraction (XRD), high-resolution transmission electron microscopy (HR-TEM), adsorption-desorption of N_2_ at −196 °C, ^13^C and ^29^Si solid state nuclear magnetic resonance (NMR), X-ray photoelectron spectroscopy (XPS) and diffuse reflectance ultraviolet-visible (DRUV-vis) spectroscopy. Titanium was present mainly in the form of titania aggregates, but also as small particles interacting with the SBA support. In both catalysts, the metal alloy nanoparticles displayed an average size of 4 nm as demonstrated by TEM measurements. AuCu/Ti-SBA turned out to be photoactive and selective in the photo-CO-PROX reaction showing the highest activity, with conversion and selectivity towards CO_2_ of 80%, due both to the presence of titania incorporated in SBA-15 and to the synergistic effect of Cu when alloyed with Au.

## 1. Introduction

Since Fujishima and Honda discovered the potential of TiO_2_ in water splitting under ultraviolet (UV) irradiation back in 1972 [1], photocatalysis has gained the interest of the scientific community worldwide. Nowadays, photocatalysis research can be broadly classified into two categories: energy and environment; the latter being extensively studied for pollutant degradation and environmental remediation of water and air, inasmuch as these reactions can be accomplished at mild operating conditions and can be solar driven [2,3,4,5]. A recent book [6] has highlighted the current developments and future potential of the green-chemistry-oriented applications of various inorganic, organic, and hybrid photocatalysts and their importance in a broad spectrum of catalytic processes such as photocatalytic CO_2_ reduction [7], photocatalytic water oxidation [8], heteropolyacid-based heterogeneous photocatalysts for environmental application [9], photocatalysts based on 1D TiO_2_ nanotubes [10], water-splitting by photocatalytic reduction [11] and solar–chemical energy conversion by photocatalysis [12].

In this way, the *n*-type semiconductor titania [13] (which naturally exists in three different polymorphs: anatase, rutile and brookite [14]), especially its anatase phase, stands out as one of the most promising and hence studied materials for photocatalytic purposes. This is due to its numerous advantages: it is a widely available material with chemical and thermal stability, high photo-activity, low cost, optical properties, and non-toxicity [15,16,17]. Nonetheless, the use of this semiconductor as a photocatalyst is limited due to the rapid recombination rate of the photogenerated electron–hole pairs within the TiO_2_ particles and by its wide band gap (3.2 eV for anatase phase [18]), therefore UV wavelengths (λ < 387 nm) are required in order to excite an electron from the valence band (hence generating hole pairs) to the conduction band, which stands for a narrow photocatalytic region taking into account that only a very small fraction of solar light, approximately 5%, consists in UV. Otherwise, TiO_2_ applicability depends not only on the properties of titania itself, i.e., particle size distribution or crystalline structure, but also on the modification of its material host as well as on the interactions with the chemical environment [19], which allows researchers to face one of the greatest challenges in photocatalysis: the development of catalysts photoactive in the visible light scope, which stands for 43% of the incident solar spectrum.

To improve the activity of TiO_2_ by modifying its morphology, one of the most studied strategies comprises the use of siliceous mesoporous materials such as MCM-41 or SBA-15 for obtaining small particle-size TiO_2_ species and, therefore, a high surface area, as several studies have demonstrated [5,20,21]. Among these two supports, SBA-15 arises as an effective one thanks to its hydrothermal stability [22] whereas MCM-41 shows poor wetting stability in aqueous solutions and has thinner wall thickness [23], which reduces confinement strength. Moreover, the combination of titania and SBA-15 has shown an enhancement in active metal dispersion in addition to surpassing the low metal-support interaction of SBA-15 and low surface area of titania [24].

The synthesis method for the introduction of TiO_2_ species rises as a crucial step for obtaining highly active and stable photocatalysts. Concerning direct synthesis, Ti incorporation into the SBA-15 framework has been successfully accomplished by hydrothermal and microwave-assisted methods [25,26]. On the other hand, different approaches for post-synthesis Ti incorporation in mesoporous silicas, such as chemical grafting and incipient wetness impregnation, have been proved as feasible alternatives to accomplish TiO_2_ species on the support external surface [27,28], but also in the pore channels, causing pore blockage when using high Ti/Si ratios, which leads to the reduction of photocatalytic activity [21]. In order to avoid this blockade, Shindo et al. [21] have developed a post-synthesis method with which a high dispersion of TiO_2_ species have been achieved maintaining the SBA-15 characteristic mesoporous structure.

TiO_2_ photoactivity can also be improved by reducing its band gap using both metal and non-metal elements. Noble metal nanoparticles such as Au, Ag, Pd or Pt have arisen as excellent means to enhance photocatalytic activity thanks to their outstanding properties. Their high optical absorption in a wide range of solar light, together with their reactivity at low temperatures and low cytotoxicity makes them suitable candidates for a new generation of proficient sunlight photocatalysts for environmental applications [29,30]. Likewise, in recent years, their remarkable localized surface plasmon resonance (LSPR) properties have increased recognition of them as harvesters of photon energy and as effective electron traps, boosting interfacial charge transfer and, therefore, hampering recombination of the electron-hole pair in chemical reactions [31,32,33,34]. This is probably due to charge transfer from the photoexcited metal to the semiconductor along with surface plasmonic resonance (SPR)-induced electromagnetic fields in plasmonic nanostructure nearness [35]. 

Au is considered as a very stable and catalytically inert metal. Nonetheless, it has been the most studied one among all noble metals since Haruta demonstrated the unexpectedly high activity of Au nanoparticles in CO oxidation at low temperature by virtue of particle size manipulation and metal-support interaction architecture modification [36]. In fact, this discovery has led to a great increase in its use in catalysis, especially in the preferential oxidation of CO in hydrogen-rich streams (CO-PROX) [37,38,39,40,41,42], as this reaction can be carried out at mild operating conditions (atmospheric pressure and temperatures in the range 40–200 °C [43,44]). This reaction can find its main application in the purification of fossil fuels produced or water–gas shift produced hydrogen so that it can be used as fuel for proton exchange membrane fuel cells (PEMFC), since even trace amounts of CO lead to platinum electrode poisoning, thus lessening the efficiency [45,46,47]. PEMFCs are deemed as a power source for transport applications, given their relatively low operation temperatures (80 °C), high efficiency and negligible emissions of regulated pollutants [48]. Oxygen adsorption and activation are rate-limiting steps for the CO-PROX reaction over Au/Ti catalysts because of oxygen dissociation being inhibited on single gold crystals [49]. Therefore, several studies suggested that this is believed to occur on the support or at the metal-support interface [37,50]. Another attainable way to accomplish oxygen dissociation comprises gold alloys with a second metal with capability to adsorb and activate oxygen. Liu et al. [51,52] demonstrated a synergistic effect between Au and Ag in CO oxidation; however, a major drawback was intrinsic to the synthesis method, which produced uncontrolled and large particle sizes. 

With the aim of developing sustainable and economic catalysts, Au doped-transition metals such as Fe, Cu, Co or Ni catalysts have gained great interest thanks to their abundance and affordability. Moreover, they have shown a synergistic effect in the degradation of pollutants and CO oxidation [44,53]. This fact can be understood considering that the incorporation of transition metals in titania may generate new energy levels between valence and conduction bands and, therefore, induce a shift of light absorption towards the visible light region [17]. 

The behaviour of bimetallic catalysts on oxidic supports is quite complex, depending on the type of metals and oxides considered. The structure of bimetallic particles on refractory oxides depends on the considered metal pair, usually a base metal from the group of Mn, Fe, Co and a noble metal [54]. During a reduction treatment, necessary to obtain a bimetallic system, the presence of a base metal is known to lower the reduction temperature of the more noble metal. Yet in these systems the full reduction of the less noble metal is not assured, leaving it in a partially oxidized state. This has been attributed to the strong oxyphilic interaction of the base metal with the refractory oxide, forming a monolayer thick oxide over the support that screens a direct interaction between the surface noble metal and the support. The less interactive noble metal, usually Pd, Pt, Rh, Ru, Ir, is found at the top of this layer, dominating the catalytic gas/solid interface and, as a consequence, the catalytic behaviour. The postulated model can be applied to several base metal/noble metal systems, nevertheless systems comprised of two noble metals on a refractory oxide will form structures predicted for bulk metal alloys [54]. When both metals belong to the coinage metal group, Ib, other factors can influence the structure more strongly than support/metal interactions, such as the composition of the reaction mixture [55]. In the present work, the bimetallic pair is composed of Au and Cu species and the matrix used as a support is based on a refractory oxide, silica, and a reducible one, titania, the latter being the photoactive substrate.

As far as we are concerned, AuCu bimetallic catalysts supported on Ti-modified SBA-15 have not yet been characterized nor tested in a CO-PROX reaction under simulated solar light (Photo-PROX). Our previous work [56] has shown a remarkable improvement in photo CO-PROX using Au/TiO_2_ catalysts with a very low Au loading (0.5 wt %), and has also demonstrated the relation between photocatalytic activity and Au particle size.

The present work sets out to analyse the photocatalytic activity of AuCu catalysts supported on ordered mesoporous SBA-15 and Ti-SBA-15 in the preferential CO photo-oxidation in hydrogen-rich streams, at atmospheric pressure and ambient temperature, assessing not only the role of solar light irradiation but also the photocatalytic performance of an AuCu bimetallic system and its electronic interaction with titania.

## 2. Materials and Methods

### 2.1. Synthesis of SBA and Ti-SBA

SBA-15 mesoporous silica was prepared according to the method reported by Cazalilla et al. [57]. This is a low-cost method that does not require expensive alkoxides as Si source. Ti-SBA mesoporous silica with a nominal Si/Ti molar ratio equal to 3 was prepared by following the procedure reported by Shindo et al. [21], where Ti was incorporated post-synthesis.

### 2.2. Preparation of Au and AuCu Supported Nanoparticles

The incorporation of Au and Cu into SBA and Ti-SBA has been carried out by following the procedure described by Liu et al. [44]. Firstly, the support surfaces were functionalized with aminopropyltriethoxysilane, APTES (NH_2_(CH_2_)_3_Si(OEt)_3_), according to the procedure described by Tu et al. [58].

Au was firstly incorporated by adding the desired amount of tetrachloroauric acid (HAuCl_4_) solution in water-dispersed APTES functionalized samples. After stirring for 2 h, samples were filtered, water washed, re-dispersed, reduced with sodium boron hydride (NaBH_4_), recovered by filtration and dried overnight at 60 °C to give Au-APTES-SBA or Au-APTES-Ti-SBA. Then, the incorporation of Cu was carried out by adding the desired amount of Cu(NO_3_)_2_ solution to a water-redispersed Au-APTES functionalized sample and stirred for 2 h before a new NaBH_4_ reduction. Finally, the samples were dried overnight and the organic part was removed calcining at 500 °C for 6 h. In order to obtain AuCu alloy, the samples were further reduced in H_2_ at 500 °C for two hours (AuCu/SBA and AuCu/Ti-SBA samples). In order to obtain Au-Cu bimetallic sample (Au-Cu/Ti-SBA), no hydrogen treatment was required. The amount of precursor salts was calculated to obtain Au catalysts with a loading of 1.5 wt % of Au, a total metal loading of 2.0 wt % and an Au/Cu molar ratio of 1.

### 2.3. Characterization of Catalysts

X-ray powder diffraction (XRPD) patterns of fresh catalysts were collected on a PAN analytical X’Pert Pro automated diffractometer. Powder patterns were recorded between 0.5° and 65° in 2θ, with a step size of 0.0167° (2θ) and an equivalent counting time of ~60 s/step, in a Bragg–Brentano reflection configuration by using a Ge (111) primary monochromator (Cu K α1) and the X’Celerator detector.

N_2_ physisorption measurements were performed at −196 °C with an ASAP 2010 apparatus of Micromeritics. Before each measurement, the samples (0.1 g) were outgassed first at 130 °C for 12 h at 0.67 Pa and then at room temperature for 2 h at 1 × 10^−4^ Pa. The N_2_ isotherms were used to determine the specific surface areas through the BET equation (S.A._BET_), and the specific pore volume (Vs) calculated at P/P_0_ = 0.98. The pore size distribution was calculated according the NLDFT (non-local density functional theory) method.

Size and morphology of the nanoparticles were studied by high-resolution transmission electron microscopy (HR-TEM) using a TALOS F200x instrument (Thermo Fisher Scientific, Waltham, MA, USA). TEM analysis was performed at 200 kV and 5.5 µA and scanning transmission electron microscopy (STEM) with a high-angle annular dark-field imaging (HAADF) detector, at 200 kV and 200 nA. Image J (ImageJ 1.51 K, National Institutes of Health, Bethesda, MD, USA) software was used to calculate the average particle size distribution.

^29^Si and ^13^C solid state nuclear magnetic resonance (NMR) analyses were carried out using the Hpdec (cw decoupling sequence) and CP-MAS technique, respectively, at a rotation speed of 15 kHz on a DVT probe of 2.5 mm of triple resonance and double wideband range and registered. ^29^Si spectra were measured with 4000 scans, D1 = 5 s and P15 = 2 ms. ^13^C spectra with an excitation pulse of 4.0 μs and a recycling time of 50 s and 2.500 scans.

The diffusive reflectance UV-vis (DRUV-vis) spectra were collected with a Perkin Lambda 35 UV-vis spectrophotometer (PerkinElmer, Waltham, MA, USA), equipped with integrating sphere accessory with the wavelength ranging from 300 to 800 nm. The absorption coefficient (α) was calculated as follows: α = ln(1/T)/d, where T is the measured transmittance and d is the optical path length. Band gap energy, Eg, was determined thoroughly the α value (m^−1^) from a plot of (αhν)^1/2^ versus photon energy (hν), where h is Planck’s constant and ν is the frequency (s^−1^). The intercept of the tangent to the absorption curves was used to estimate the band gap (Eg) value.

X-ray photoelectron spectra (XPS) were collected using a Physical Electronics PHI 5700 spectrometer (Physical Electronics, Inc., Chanhassen, MN, USA) with non-monochromatic Mg Kα radiation (300 W, 15 kV, 1253.6 eV) for the analysis of the core level signals of C 1*s*, O 1*s*, Ti 2*p* and Au 4*f* with a multi-channel detector. Binding energy (BE) values were referenced to the C 1*s* peak (284.8 eV) from the adventitious contamination layer. The spectrometer energy scale was calibrated with plasma etched Cu, Ag and Au foils using Cu 2*p*_3/2_, Ag 3*d*_5/2_, and Au 4*f*_7/2_ photoelectron lines at 932.7, 368.3, and 84.0 eV, respectively. The PHI ACCESS ESCA-V6.0 F software package and Multipak v8.2b were used for acquisition and data analysis, respectively. A Shirley-type background was subtracted from the signals. Recorded spectra were always fitted using Gauss-Lorentz curves, in order to determine the binding energy of the different element core levels more accurately. The error in BE was estimated to be ca. 0.1 eV.

### 2.4. Photocatalytic Activity in CO-PROX

CO-PROX catalytic tests were carried out in a laboratory flow apparatus with a fixed bed reactor operating at atmospheric pressure. The catalyst (0.15 g) was placed in a quartz cell with a cooling water system. The gas hourly space velocity, GHSV, was 22,000 h^−1^. The feed consisted of 1.25% CO, 1.25% O_2_ and 50% H_2_ (vol. %) balanced with He. The temperature of the quartz cell was controlled at about 30 °C (measured by a thermocouple placed inside the catalyst bed). During the testing process, a visible light (Sunlight Solar Simulator, AM1.5G filter, 100 watt Xenon arc lamp, Abet Technologies, Milford, CT, USA) was introduced into the surface of the quartz cell. For testing the thermocatalytic activity of catalyst under dark, the quartz cell was wrapped with Al foils to shut down light irradiation.

The carbon monoxide and oxygen conversions were calculated based on the CO Equation (1) and O_2_ Equation (2) consumption, respectively:

(1) $$CO\;Conversion\;\left(\%\right)=\frac{n_{CO}^{in}-n_{CO}^{out}}{n_{CO}^{in}}\times 100$$

(2) $$O_{2\;}Conversion\;\left(\%\right)=\frac{n_{O_{2}}^{in}-n_{O_{2}}^{out}}{n_{O_{2}}^{in}}\times 100$$

The selectivity towards CO_2_ was estimated from the oxygen mass balance as follows Equation (3):

(3) $$Selectivity\;\left(\%\right)=\frac{n_{CO}^{in}-n_{CO}^{out}}{2\times \left(n_{O_{2}}^{in}-n_{O_{2}}^{out}\right)}\times 100$$

The excess of oxygen factor (λ) Equation (4) used was 2 because this value was previously found optimal for CO-PROX [59]:

(4) $$\lambda =2\times \frac{n_{O_{2}}^{in}}{n_{CO}^{in}}$$

## 3. Results

### 3.1. Characterization of Supports

**Figure 1:**
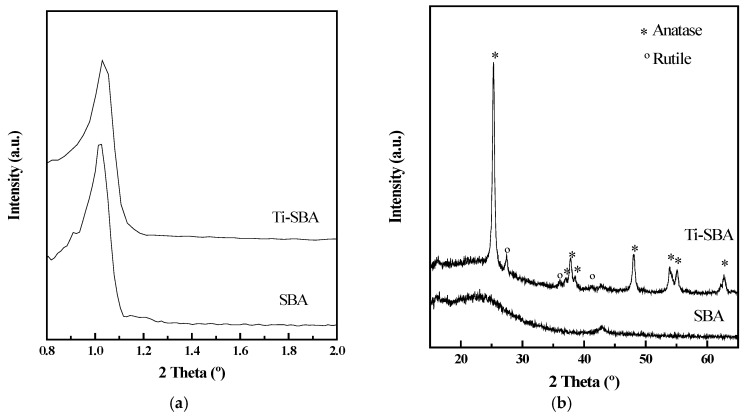
(**a**) Low-angle and (**b**) high-angle X-ray diffractograms of the support material.

X-ray diffractograms of the supports are presented in Figure 1. Figure 1a shows the profiles at low angles, where a diffraction peak at 2θ between 1.01° and 1.09° can be observed, corresponding to d_100_ reflection for hexagonal symmetry typical of ordered mesoporous silica. It can be noticed how after Ti incorporation, a slight shift of the maxima is observed, corresponding to a decrease of the d_100_ parameter from 8.74 to 8.57 nm. This suggests that part of the Ti could be incorporated in the mesoporous structure.

XRD profiles at high angles exhibit in both cases a broad signal between the 20–30° characteristic of amorphous materials like SBA-15. Ti-SBA support shows the presence of several diffraction peaks ascribed to the presence of anatase and rutile phases. The main signals are those associated to anatase (PDF No.: 01-089-4921) at 2θ (°) = 25.3, 37.0, 37.8, 38.6, 40.1, 53.9, 55.1 and 62.8. Meanwhile, the diffraction peaks at 2θ (°) = 27.4, 36.0 and 41.3, correspond to rutile polymorph (PDF No.: 01-072-1148). Semi-quantitative analysis by using HighScore Plus programme from P’Analytical (Almelo, The Netherlands) indicates that 86% of TiO_2_ is anatase and 14% rutile. On the other hand, the estimated anatase mean crystal size, calculated by using Scherrer equation, was approximately 24 nm.

**Figure 2:**
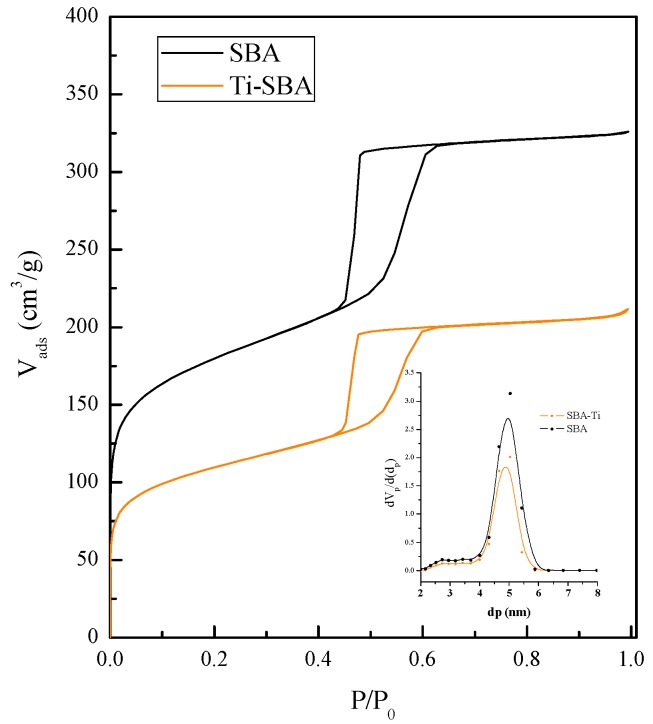
N_2_ adsorption–desorption isotherms at −196 °C of bare supports.

The main textural features of bare supports from the appropriate treatment of N_2_ adsorption–desorption data are summarized in Table 1 and their isotherms and pore size distributions are represented in Figure 2. In the case of SBA-15 silica support, a type IV isotherm with type H1 hysteresis loop in the IUPAC classification, which is characteristic of mesoporous SBA-15 type materials, was observed. The BET specific surface area and the cumulative pore volume are 654 m^2^ g^−1^ and 0.50 cm^3^ g^−1^, respectively, suggesting relatively large internal pore surface. The pore size distribution, depicted in the inset of Figure 2, is quite narrow and located in the mesopore region, with a mean pore diameter centered at 5.0 nm. The wall thickness of the SBA was estimated to be 5.1 nm. As expected, the insertion of titanium dioxide in the SBA sample provoked a decrease of specific surface area, pore volume and pore size from 654 to 393 m^2^ g^−1^, with a concomitant decrease of total pore volume from 0.50 to 0.32 cm^3^ g^−1^. The pore size distribution of SBA-Ti remained very similar after the incorporation of titania, passing from 5.0 to 4.8 nm. It can be suggested that the presence of titanium dioxide did not alter the mesoporous nature of the material; in fact, the wall thickness remained unaltered, but the lower amount adsorbed at low relative pressures indicates the filling of pores.

**Figure 3:**
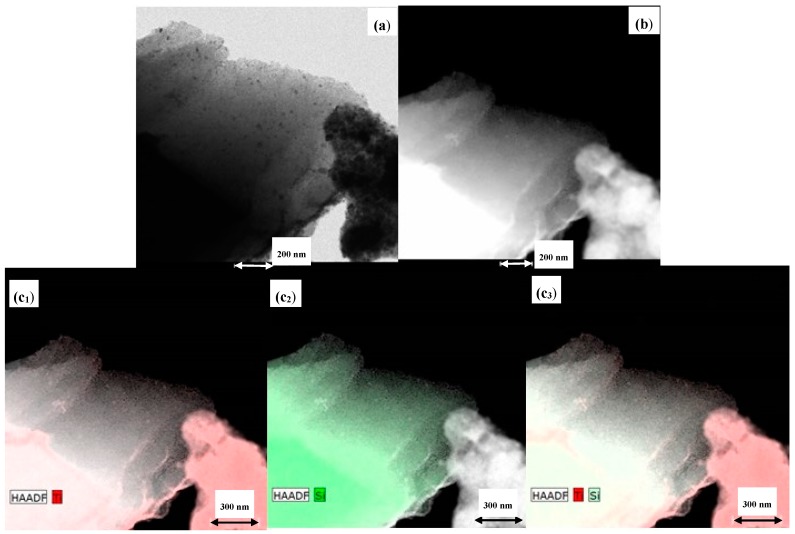
(**a**) Transmission electron microscopy (TEM), (**b**) scanning transmission electron microscopy (STEM) and (**c_i_**) mapping results of the Ti-SBA support.

The preferential location of titania on the SBA-15 material support was elucidated by HR-TEM. The corresponding micrographs are included in Figure 3. First, the well-ordered structure of the SBA-15 mesoporous sample is clearly evident. Titanium oxide is present as agglomerates of relatively large size, but also small titania nanoparticles are present in close contact with the SBA support and located inside the porous channels as mapping results of the Ti-SBA support reflect.

**Figure 4:**
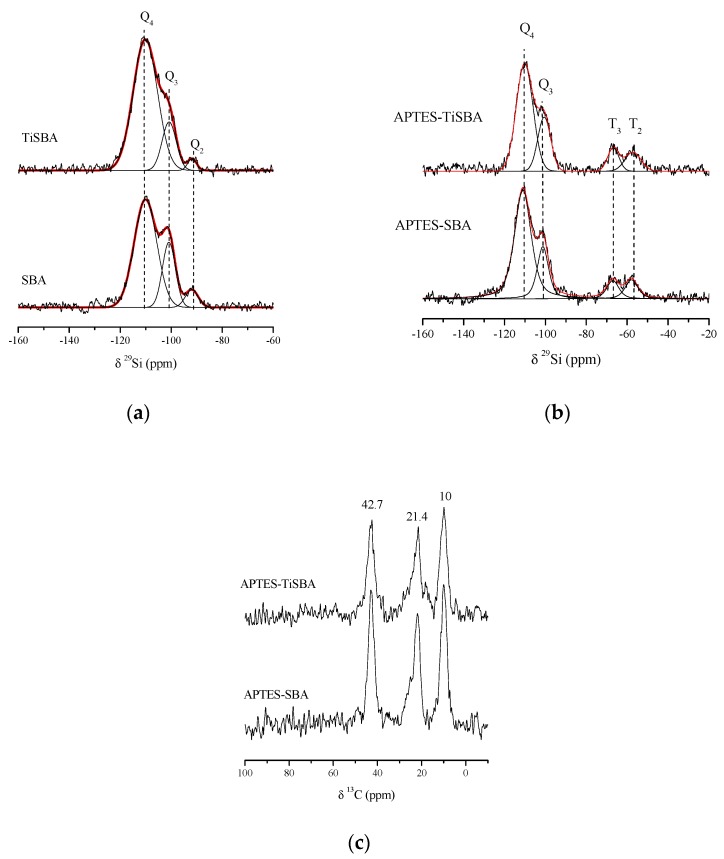
(**a**) ^29^Si nuclear magnetic resonance (NMR) spectra of bare supports, (**b**) ^29^Si NMR spectra and (**c**) ^13^C NMR spectra of APTES functionalized SBA and Ti-SBA.

In order to obtain further information about the incorporation of Ti and how silica coordination was altered, solid state ^29^Si NMR spectra were recorded (Figure 4a).

The ^29^Si NMR spectrum of pristine SBA-15 shows three contributions corresponding to tetra-functional silicon centers, Q*_n_*:(SiO)*_n_*Si(OX)_4−*n*_, where *n* refers to bridging oxygen atoms surrounding the central silicon atom and X = H. Thus, Q_4_, Q_3_, and Q_2_ resonances at ca. −110, −102, and −91 ppm, refer to [Si(OSi)_4_], [Si(OSi)_3_OH] and [Si(OSi)_2_(OH)_2_] silicon sites, respectively [60,61]. The relative populations of silicon environments were calculated by deconvolution of the spectra into individual Gaussian peaks by using DMfit software and the corresponding relative peak areas are displayed in Table 2. SBA sample was mainly composed of Q_4_ sites, but also sites providing OH groups (Q_3_ and Q_2_) were present. After Ti incorporation, it is clearly observed how Q_2_ contribution disappears and the proportion of Q_3_ sites decreases. These data point out the preferential interaction of Ti with OH sites on the silica surface.

The chemical grafting of APTES to the silica frameworks was investigated by means of ^29^Si and ^13^C measurements and displayed in Figure 4b,c, respectively. ^29^Si spectra show the presence of tetra-functional silicon centers, as described before, but also, new resonance bands due to the appearance of tri-functional silicon centers, T*_m_*, where T refers to [(SiO)*_m_*RSi(OX)_3−*m*_] units and m being the number of bridging oxygen atoms surrounding the central silicon atom. T_3_ and T_2_ sites can be found at −67 ppm and −57 ppm, respectively, which are attributable to T_3_ [(SiO)_3_R], and T_2_ [(SiO)_2_RSiOH] units. These T species confirm that some hydroxyl groups, initially at the Q_2_ and Q_3_ silicon atoms, have reacted by covalent bonding with APTES. The results of deconvolution clearly reflect the disappearance of Q_2_ sites and the decrease of Q_3_ ones.

On the other hand, ^13^C CP-MAS spectra also presented signals at ca. 10.0, 21.4, and 42.7 ppm assigned to the presence of –Si–CH_2_–, –CH_2_–CH_2_–, and –CH_2_–NH_2_ groups, respectively, from APTES molecules. At first glance, both samples present similar intensities in the signals once normalized by sample mass, what suggests that Ti incorporation does not alter the surface functionalization with APTES.

### 3.2. Characterization of Photocatalysts

**Figure 5:**
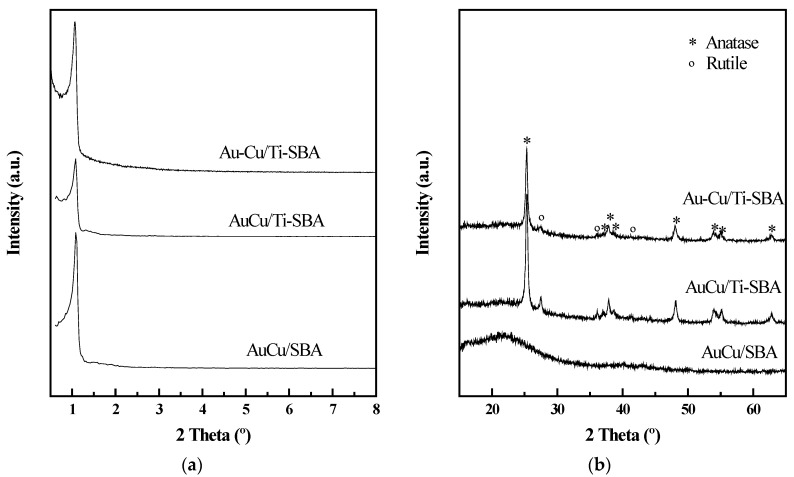
(**a**) Low-angle and (**b**) high-angle X-ray diffractograms (XRD) for the as-prepared samples.

X-ray diffractograms of the catalysts are included in Figure 5. Low-angle diffractograms (Figure 5a) presented d_100_ reflection; an indication of the maintenance of a mesoporous structure [62]. However, the maximum is shifted to higher angles after AuCu incorporation. This is indicative of the preferential location of metallic particles inside the porous structure, as observed by others [32]. In absence of Ti, d_100_ values are smaller, pointing to a greater number of metallic particles inside the channels than when titanium is present in the catalyst formulation. This could be explained by the high Ti loading present (30 wt % ca.) in the samples, as part of the pores could be blocked and therefore hindering the entrance of the metallic particles inside. TEM results corresponding to Ti-SBA confirm this fact. At high angles, AuCu/SBA did not present any diffraction peak, but Ti-SBA based samples show the presence of the diffraction peaks observed for the material support and ascribed to anatase and rutile. The estimated anatase mean crystal size hardly changed after metal deposition.

The textural properties changed after metal incorporation as it can be observed in Table 1, although the structure was maintained in all three cases as evidenced by XRD, the maintenance of Type IV isotherm and TEM results. As will be discussed below, the presence of small nanoparticles mainly located inside the channels could be responsible for such a fact. If SBA and Ti-SBA based catalysts are compared, SBA is affected substantially more by metal incorporation. This could be explained considering that some metal particles are in close contact with titania agglomerates, so not all the metallic phase is interacting with SBA support and thus its surface suffers a minor decrease.

**Figure 6:**
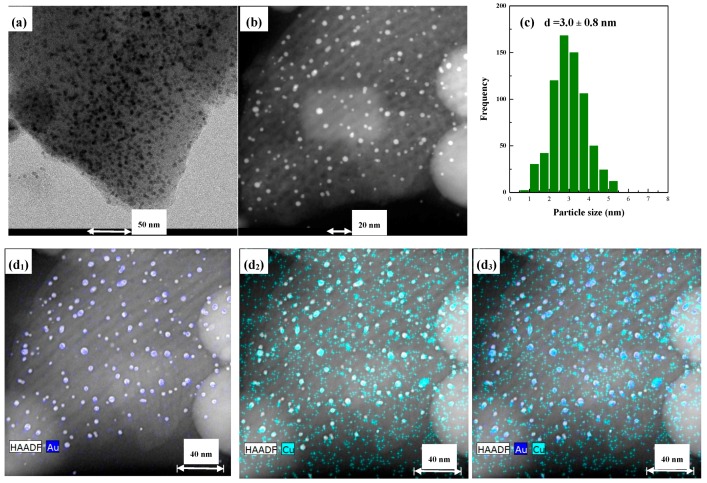
(**a**) TEM, (**b**) STEM, (**c**) particle size distribution and (**d_i_**) mapping results for AuCu/SBA sample.

**Figure 7:**
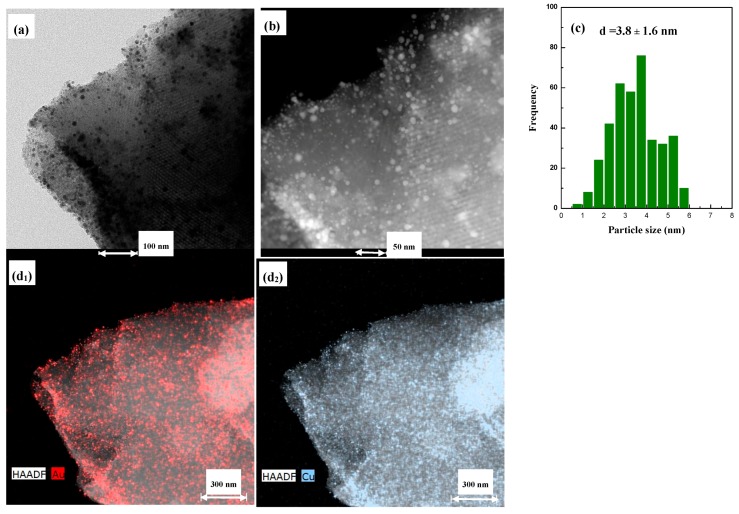
(**a**) TEM, (**b**) STEM, (**c**) particle size distribution and (**d_i_**) mapping results for AuCu/Ti-SBA sample.

**Figure 8:**
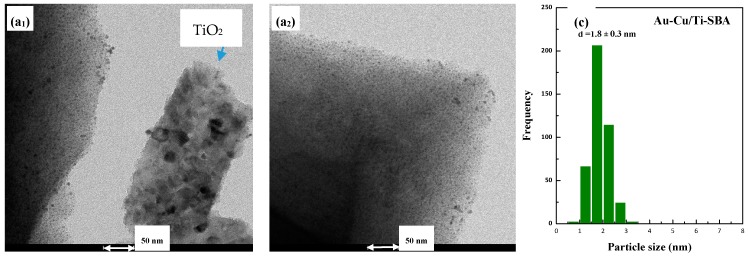

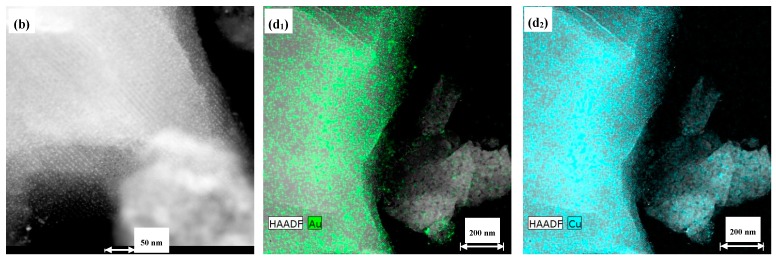
(**a_i_**) TEM, (**b**) STEM, (**c**) particle size distribution and (**d_i_**) mapping results for Au-Cu/Ti-SBA sample.

TEM micrographs (Figure 6, Figure 7 and Figure 8) of the as-prepared samples provided an approximation of the metallic distribution onto the support microstructure. In all cases it was observed the regular ordered structure of the mesopores corresponding to SBA-15 mesoporous silica corroborating the maintenance of mesoporous structure after Ti and Au-Cu incorporation. Moreover, it is evidenced that the metallic particle size is strongly dependent on the catalysts composition and either the presence or absence of an alloy. Considering AuCu/SBA catalyst, this presents a homogeneous distribution of metallic particles all over the support. STEM micrographs (Figure 6) evidence the preferential location of the particles inside the pore structure. An estimation of the particle sizes evidenced that the mean particle size for this sample was 3.0 nm. Moreover, mapping results revealed that Au and Cu were in close contact.

The incorporation of Ti into the SBA material affected the particle size attained. Figure 7 compiles the microscopic characterization for the AuCu/Ti-SBA sample. At first glance, the particle size is not as homogeneous as it was in the absence of Ti. In the sample, small and bigger particles coexist, although their size was always lower than 6–7 nm. The corresponding histogram confirms this fact, where the mean particle size calculated was 3.8 nm, not far from that of AuCu/SBA, but the deviation is considerably higher: 1.6 vs. 0.8 nm. Mapping results again showed that Au and Cu are close and homogenously distributed on the support.

Finally, in the case of the sample not post-treated in hydrogen, Au-Cu/Ti-SBA, where it is expected that the alloy has not been formed, the presence of very small particles interacting with both SBA and TiO_2_ aggregates is observed. The estimated particle diameter evidenced a narrow distribution of particle sizes, centered at 1.8 nm. A homogeneous distribution of both Au and Cu elements on the support is also observed.

XPS measurements were performed to obtain further information about the surface composition, the chemical states of the different species on the surface as well as to elucidate interactions among the different components on the catalyst surface.

**Figure 9:**
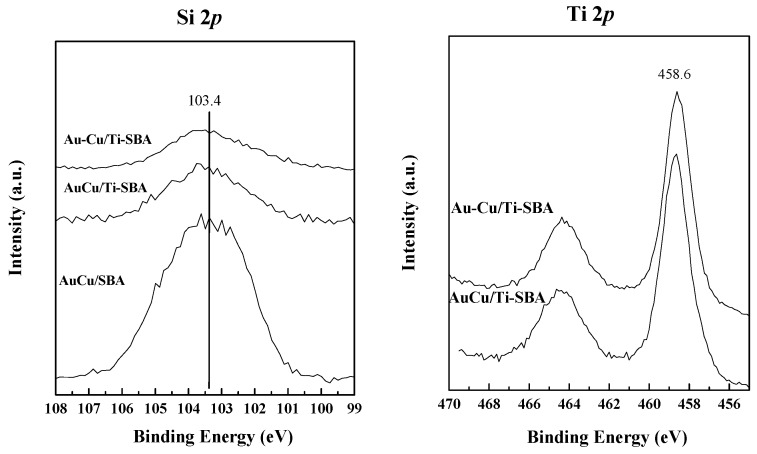

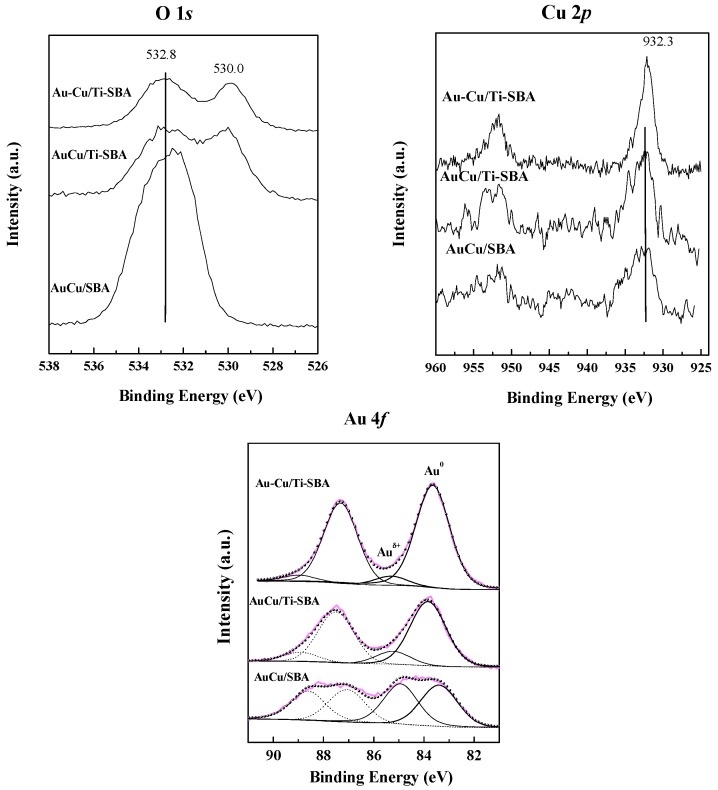
Si 2*p*, O 1*s*, Ti 2*p*, Cu 2*p* and Au 4*f* core-level spectra of the prepared samples.

Si 2*p* signal (Figure 9) shows a main peak at a binding energy of 103.4 eV, which corresponds with SiO_4_ bond state in SiO_2_. This fact is in agreement with ^29^Si NMR results discussed above, where the major contribution was assigned to Q_4_ [Si(OSi)_4_]. When Ti is present, the intensity of the signal decreases substantially as expected due to the covering of SiO_2_ by titania species. Regarding O 1*s* spectra, all of them presented a peak at 532.8 eV approximately related to the silica substrate [63]. In the case of samples with Ti, a second contribution at ca. 530.0 eV is observed due to oxygen in the TiO_2_ crystal lattice, as reported [64]. The Ti 2*p* spectra presents a main peak located at binding energy 458.6 eV ca. (Ti 2*p*_3/2_), which is ascribed to Ti^4+^ species on the catalyst’s surface.

The Cu 2*p* signal was quite noisy. It should be considered here that Cu loading in all samples was no more than 0.5 wt %, thus, along with the short exposition time of the measurements in order to avoid Cu reduction, signal noise impeded its deconvolution. Furthermore, the Cu*_LMM_* signal is very weak and noisy due to the low Cu loading and the short irradiation time. Nonetheless, a peak at average binding energy 932.1 eV (Cu 2*p*_3/2_) can be noticed, which could be attributed to Cu^0^ and Cu^1+^ species, as both are very close in binding energy [65]. The 20 eV difference among the doublet corresponding to Cu 2*p*_1/2_, located at 952.1 eV, and the one of the Cu 2*p*_3/2_ contribution, suggests the coexistence of both Cu^0^ and Cu^1+^ species, with a greater contribution of the latter [66], but with a shift to lower binding energy, possibly due to electron transfer from Au to Cu in the alloy [53]. This electron transfer, although against Pauling’s electronegativity table, has been previously reported for AuCu alloy as other factors such as the chemical state of surface elements could lead in a deficit electron state for Cu and hence reversing the expected electron transfer [67]. Wang et al. [52] also observed this phenomenon in Au alloy with Ag, which has similar electronegativity to Cu. This decrease in binding energy could be associated to a stronger electron transfer from Au to Cu, remaining the latter partially negatively charged as Cu^δ−^. Despite the noisy signal, both samples reduced at high temperature (AuCu/SBA and AuCu/Ti-SBA) present a Cu^2+^ signal contribution at 934.0 eV together with weak satellites at higher binding energies, indicating the presence of Cu^2+^. In the Au-Cu/Ti-SBA sample, reduced at milder conditions (NaBH_4_ reduction), no Cu^2+^ signal was observed, which can be explained considering the very small particle size attained (~2 nm) and the great dispersion seen in HR-TEM images, making copper species more susceptible to reduction with the X-ray irradiation beam [68].

Finally, Au 4*f*_5/2_ signals have been decomposed for the three samples. Both Au 4*f*_7/2_ (solid lines) and Au 4*f*_5/2_ (dotted lines) spin-orbit components are displayed Au spectra in Figure 9, with a Full Width at Half Maximum (FWHM) of ca. 1.6 eV. For the catalyst without titania, AuCu/SBA, the peak attributed to gold in its metallic state, Au^0^, is located at 83.4 eV, 0.6 eV lower than the expected one, 84.0 eV [69]. Also, a significant contribution of Au^δ+^ species is observed as a consequence of electronic interaction between Au and support [56] and also because of electron transfer from Au to Cu, as discussed in Cu 2*p* spectra. In samples containing Ti, Au^δ+^ contribution is less noticeable and a shift to a close value of binding energy expected for Au^0^ is observed (83.8 eV), suggesting that the formation of Au^0^ is favored in the presence of titania, due to electron transfer from oxygen vacancies of the TiO_2_ [70], which supports TEM results where it can be clearly observed that Au are in close contact with titania confined inside SBA structure but also with titania agglomerates.

Finally, the signal for the non-alloyed sample shows a peak at a binding energy very close to the one without Ti but with a very small contribution of Au^δ+^ species. This fact, indeed, asserts the formation of the alloy AuCu, as in this case no electron transfer is taking place from gold to copper and, therefore, a higher proportion of Au on the surface is interacting with titania and SBA, leading to the formation of Au in its metallic state.

Surface atomic ratios among Au, Cu and Si + Ti have been calculated and are reported in Table 3. Regarding (Au + Cu)/(Si + Ti) ratio, it is easily observed that Cu incorporation, as well as titania’s, increases the surface concentration of the bimetallic phases. In the case of the non-reduced sample, the surface atomic ratio for Au with respect to the support elements is twice the one for its reduced counterpart, and in reverse for Cu, which is consistent with TEM images where a higher dispersion of both Au and Cu can be clearly observed for the non-reduced sample. Analyzing Au/Cu ratios, it seems that in samples treated with hydrogen after their calcination, Cu could be decorating Au nanoparticles, therefore reducing Au surface exposition and increasing Cu/(Si + Ti) ratio.

**Figure 10:**
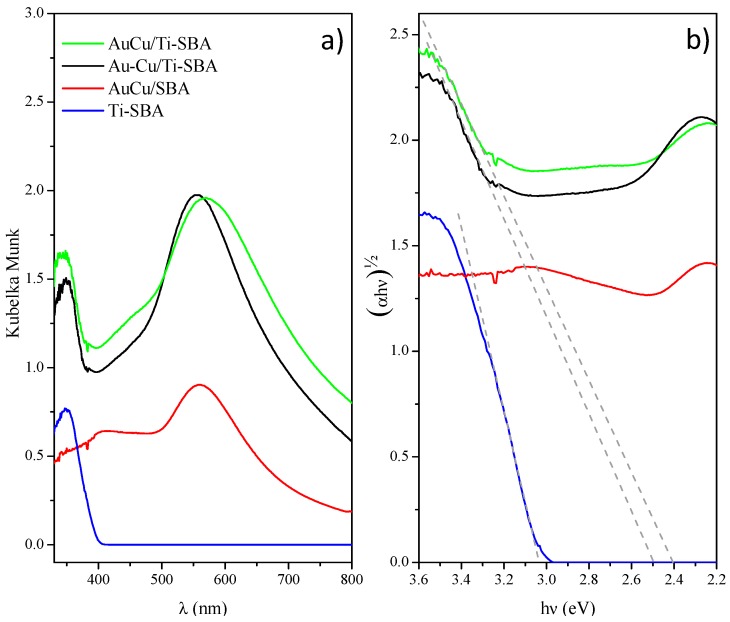
(**a**) Diffuse reflectance ultraviolet-visible (DRUV-vis) spectra of the samples and (**b**) (αhν)^½^ vs. photoenergy (hν).

The photo-response of the prepared catalysts was investigated by DRUV-vis spectroscopy and spectra of Ti-SBA, AuCu/SBA, Au-Cu/Ti-SBA and AuCu/Ti-SBA samples are shown in Figure 10. In all the spectra containing titania, an absorption peak in the UV region below 400 nm was assigned to the intrinsic absorption of TiO_2_ and in the case of the samples with metal nanoparticles (NPs) a broad band in the visible region, located between 450 and 750 nm, due to the SPR phenomenon was also detected (Figure 10a).

The SPR peak generated by the incident visible radiation on a metallic nanoparticle has generally a position and an intensity that depend both on shape and size of metal NPs as well as on the dielectric constant of the surrounding medium [71]. The Au-Cu/Ti-SBA sample shows a large SPR peak centered at about 555 nm, suggesting the presence of very small Au nanoparticles, as also found by HR-TEM measurements, in contact with a dielectric matrix [72]. The AuCu/Ti-SBA sample shows instead a larger SPR band centered at 570 nm with a tail at higher wavelengths ascribable to the presence of alloyed AuCu NPs on the support containing TiO_2_. Also, the AuCu/SBA sample profile shows a broader SPR peak with respect to the not Au-Cu alloyed sample, centered at about 561 nm and that takes into account the AuCu alloy finely dispersed on a matrix that does not contain any dielectric medium [73].

The optical band gap of the samples was estimated from the linear extrapolation of (αhν)^½^ versus photon energy (hν) curves, where h is Planck’s constant and ν is the frequency (s^−1^). While Ti-SBA gives a band gap value of 3.04 eV, the metal NPs containing samples exhibit quite lower values, of 2.49 eV for the sample Au-Cu/Ti-SBA and 2.40 eV for AuCu/Ti-SBA (Figure 10b).

From these experimental findings, the presence of metal NPs finely dispersed on a highly porous silica not only decreases the band gap energy of the titania, but the alloy nanoparticles enhance scattering efficiency because of the larger bandwidth, as reported in the literature [73].

### 3.3. Photocatalytic Activity in Preferential Oxidation of CO (CO-PROX) Reaction

**Figure 11:**
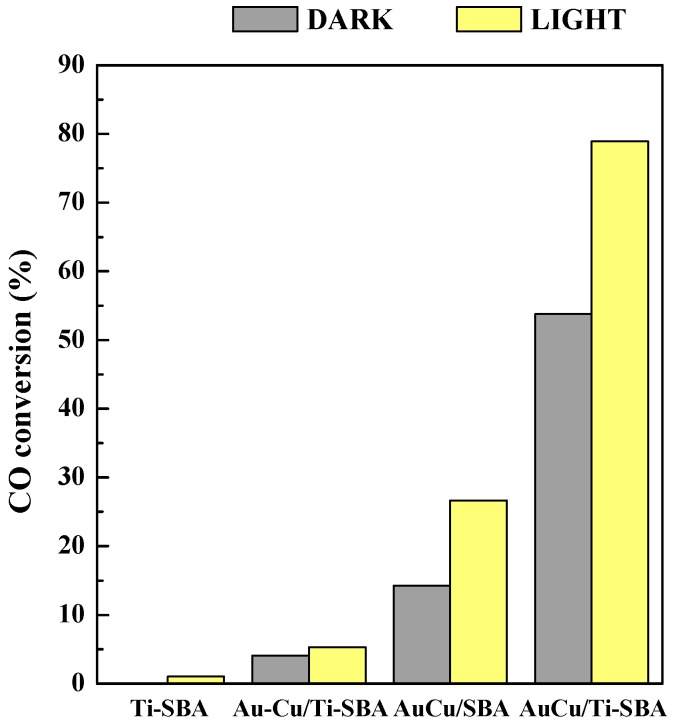
Conversion for the CO preferential oxidation in excess of hydrogen in dark and simulated solar light irradiation.

**Figure 12:**
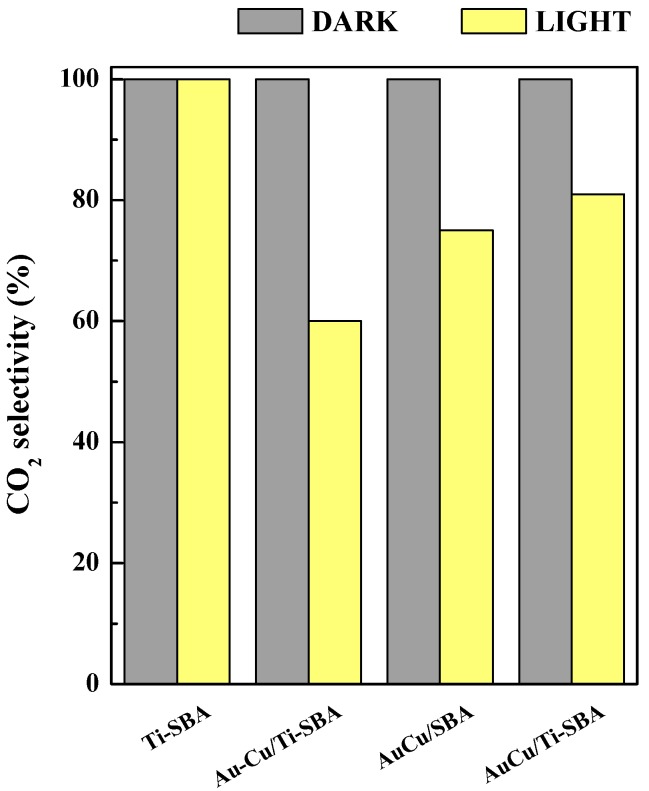
CO_2_ selectivity for the CO preferential oxidation in excess of hydrogen in dark and light mode.

Figure 11 and Figure 12 show the performance of the studied catalysts in the CO preferential oxidation in excess of hydrogen at room temperature and atmospheric pressure under dark and simulated solar light irradiation. The SBA pure silica support does not show any activity, neither in dark or light conditions (not shown), whereas the Ti-SBA matrix exhibits a negligible photoactivity, of about 1% (Figure 11). The catalysts investigated are active and selective in the test reaction: in dark mode the presence of metallic NPs lends a rather good activity in oxidizing CO, while under simulated sunlight the reactivity is strongly enhanced by the presence of AuCu alloy, so that the order for the samples is the following: Au-Cu/Ti-SBA << AuCu/SBA < AuCu/Ti-SBA (Figure 11). AuCu/SBA sample shows the highest gap in conversion between dark (14%) and light (27%) modes that reaches almost 50%. AuCu/Ti-SBA catalyst is the most active and selective under solar light irradiation, with a CO conversion of 54% in the dark and 79% under light irradiation and a CO_2_ selectivity of 100% and 81%, respectively. As shown in Figure 12, under simulated sunlight, CO_2_ selectivity follows the same trend of the CO conversion, for all the catalysts analysed.

Two main phenomena have to be considered occurring at the metal NPs/support-containing-TiO_2_ interface during a photochemical reaction: Schottky barrier formation and SPR. As known [74], under light irradiation if a metal NPs/semiconductor junction is established the difference in their Fermi levels causes a Schottky barrier between the metal and the oxide, diminishing the rate of recombination between e^−^/h^+^ favouring the photocatalytic activity. In the presence of the Schottky barrier, in fact, electrons are trapped in the metal, unable to flow back to the titania. This driving force for electron transfer from TiO_2_ to Au can be attributed to the metal/oxide interface conjugation and the differential Fermi energy level of metal with oxide. Moreover, in bimetallic alloy samples the well-known decrease of the work function of the alloyed systems with respect to the metal components leads to the prevention in the recombination rate of charge carriers [74,75,76]. Besides, in our case, the SPR phenomenon induced by the incident visible light radiation on metal NPs behaves as an electron relay able to improve the CO photo-oxidation by O_2_ molecules.

In the Au-Cu/Ti-SBA sample, on the contrary, the presence of copper partially oxidized species not alloyed with gold, can hinder the electron transfer from the negatively charged Au to the 2π orbital of adsorbed O_2_.

## 4. Conclusions

AuCu bimetallic NPs catalysts supported on SBA-15 and Ti-SBA-15 mesoporous silica were synthesized, fully characterized and tested in the CO-PROX reaction under simulated solar light irradiation at ambient conditions. A Ti-SBA support prepared by a post-synthesis route showed the maintenance of the SBA-15 characteristic mesoporous structure, ensured by low-angle XRD and N_2_ physisorption data. Incorporation of the AuCu bimetallic phase was investigated by HR-TEM, revealing the presence of very small nanoparticles homogenously distributed on the supports, interacting with titania, as assessed by XPS measurements.

Despite the low metal loading (2.0 wt %), the catalysts resulted active and selective in the studied reaction. In particular, the AuCu/Ti-SBA catalyst showed the highest CO conversion (~80%) in light mode as well as the greatest selectivity towards CO_2_ both in the dark (100%) and under simulated solar light (~80%). This can be attributed to several factors, among which the synergistic effect of gold-copper NPs, finely dispersed on a highly porous silica-titania network, that not only decrease the band gap of the titania but also enhance the scattering efficiency thanks to the larger bandwidth in comparison to an Au-Cu system that is not alloyed.

The strategy here described may open a feasible route to exploit the capability of solar light to drive the CO removal in H_2_-rich streams at ambient conditions by coinage metals supported on an ordered mesoporous silica-titania matrix.

## Figures and Tables

**Table 1:** Structural and textural properties of the support and as-prepared samples.

Sample	d_100_ (nm)	S_BET_ (m^2^ g^−1^)	V_p_ (cm^3^ g^−1^)	d_p_ (nm)
SBA	8.74	654	0.50	5.0
Ti-SBA	8.57	393	0.32	4.8
AuCu/SBA	8.10	200	0.20	4.3
AuCu/Ti-SBA	8.25	237	0.19	4.3
Au-Cu/Ti-SBA	8.33	240	0.21	4.3

**Table 2:** ^29^Si NMR band position and its contribution.

SBA			APTES_SBA		Ti-SBA		APTES-Ti-SBA	
	δ (ppm)	%	δ (ppm)	%	δ (ppm)	%	δ (ppm)	%
Q_4_	−110.1	69.4	−111.1	63.1	−110.2	80.1	−110.2	58.6
Q_3_	−101.0	24.1	−101.2	20.7	−100.8	17.3	−100.6	22.8
Q_2_	−91.9	6.5	-		−91.8	2.6	-	-
T_3_	-	-	−67.0	7.7	-	-	−66.8	8.6
T_2_	-	-	−57.8	8.5	-	-	−57.2	9.9

**Table 3:** X-ray photoelectron spectroscopy (XPS) surface atomic ratios for the prepared samples.

Sample	Au/(Si + Ti)	Cu/(Si + Ti)	(Au + Cu)/(Si + Ti)	Au/Cu
AuCu/SBA	0.018	0.029	0.047	0.65
AuCu/Ti-SBA	0.035	0.070	0.105	0.49
Au-Cu/Ti-SBA	0.071	0.053	0.123	1.33
